# Analgesic Efficacy and Safety of Tapentadol Immediate Release in Bunionectomy: A Meta-Analysis

**DOI:** 10.3390/ph16091287

**Published:** 2023-09-12

**Authors:** Lorenzo Franco-de la Torre, Eduardo Gómez-Sánchez, Othoniel Hugo Aragon-Martinez, Adriana Hernández-Gómez, Diana Laura Franco-González, Juan Manuel Guzmán-Flores, Angel Josabad Alonso-Castro, Vinicio Granados-Soto, Mario Alberto Isiordia-Espinoza

**Affiliations:** 1 Instituto de Investigación en Ciencias Médicas, Cuerpo Académico Terapéutica y Biología Molecular (UDG-CA-973), Departamento de Clínicas, División de Ciencias Biomédicas, Centro Universitario de los Altos, Universidad de Guadalajara, Tepatitlán de Morelos 47620, Mexico; lorenzo.franco@academicos.udg.mx (L.F.-d.l.T.); adriana.hgomez@academicos.udg.mx (A.H.-G.); diana.franco5288@alumnos.udg.mx (D.L.F.-G.); 2 División de Disciplinas Clínicas, Centro Universitario de Ciencias de la Salud, Universidad de Guadalajara, Guadalajara 44340, Mexico; eduardo.gsanchez@academicos.udg.mx; 3 Laboratorio de Productos Naturales, Facultad de Ciencias Químicas, Universidad Autónoma de San Luis Potosí, San Luis Potosí 78210, Mexico; hugo.aragon@uaslp.mx; 4 Departamento de Ciencias de la Salud, División de Ciencias Biomédicas, Centro Universitario de los Altos, Universidad de Guadalajara, Tepatitlán de Morelos 47620, Mexico; juan.guzman@cualtos.udg.mx; 5 Departamento de Farmacia, División de Ciencias Naturales y Exactas, Universidad de Guanajuato, Guanajuato 36040, Mexico; angeljosabad@hotmail.com; 6 Neurobiology of Pain Laboratory, Departamento de Farmacobiología, Cinvestav, South Campus, Mexico City 14330, Mexico; vinicio_granadossoto@hotmail.com

**Keywords:** tapentadol, hallux valgus surgery, bunionectomy, pain control, adverse effects

## Abstract

The aim of this systematic review and meta-analysis was to evaluate the analgesic effect of different doses of tapentadol immediate release (IR) and its adverse effects after a bunionectomy. Pubmed, Cochrane, Lilacs, Medline, and Imbiomed were used to identify abstracts of scientific publications related to the keywords of this systematic review (PROSPERO ID CRD42023437295). Moreover, the risk of bias in all included articles was assessed using the Cochrane Collaboration risk of bias tool. Data on the sum of pain intensity, total pain relief, global assessment, and adverse effects were extracted. The statistical method of inverse variance with means difference was used to evaluate the numerical data and the Mantel–Haenszel and Odd Ratio test to analyze the dichotomous data. In addition, the number needed to treat, the number needed to harm, and the 95% confidence intervals were calculated. A qualitative evaluation (*n* = 2381) was carried out according to the conclusions of the authors. Tapentadol (*n* = 1772) was more effective in relieving postoperative pain than the placebo (*n* = 609) after a bunionectomy. In addition, the analgesic efficacy of IR tapentadol (*n* = 1323) versus the placebo (*n* = 390) was evaluated in a total of 1713 patients using a global evaluation of the treatments. All three doses of IR tapentadol showed better results compared to the placebo after a bunionectomy. Finally, the adverse effects have a direct relationship with the dose, and the greatest number of adverse effects are most observed with tapentadol IR 100 mg (*n* = 2381). It is concluded that tapentadol IR (100 mg) leads to the best satisfaction score in this meta-analysis.

## 1. Introduction

Postoperative pain after a bunionectomy occurs during the first 24 h post-surgery, and the patient may experience bouts of pain for several days [1,2]. This surgical procedure is considered a characterized clinical model for the evaluation of postoperative pain [1,2,3]. Therefore, a bunionectomy is a very important clinical tool for developing new analgesics or evaluating the clinical efficacy and safety profile of currently marketed drugs [1,2,3,4].

The most common postoperative analgesic treatment after bunion surgery mainly includes the use of non-steroidal anti-inflammatory agents (NSAIDs) [1] and opioid analgesic drugs [2]. In addition, various analgesic regimens can be used (e.g., pre-preventive analgesia, a combination of drugs to employ multimodal analgesia, and local analgesia) [5]. An analgesic monotherapy approach is common, and one of the most widely used opioid drugs in published clinical trials has been tapentadol immediate release (IR) [6,7,8,9,10,11,12]. However, tapentadol is a relatively new drug and limited scientific evidence of its analgesic efficacy in a bunionectomy can be found in a few individual clinical trials [6,7,8,9,10,11,12].

Based on the results of these trials, tapentadol has clinical efficacy for pain control similar to oxycodone [8,10] or morphine [11]. However, the most effective dose of tapentadol IR based on its clinical efficacy and adverse effects in a bunionectomy has not been defined. For this reason, the purpose of this systematic review and meta-analysis was to assess the analgesic effect of different doses of tapentadol IR and its adverse effects after a bunionectomy.

## 2. Material and Methods

### 2.1. Information Search

Pubmed, Cochrane, Lilacs, Medline, and Imbiomed were used to identify abstracts of scientific publications related to the keywords of this systematic review. The following search terms were used for the Pubmed, Cochrane, Lilacs, and Medline databases: “tapentadol” AND “bunionectomy”; “tapentadol” AND “hallux valgus surgery”; “tapentadol” AND “orthopedic surgery”; “µ-opioid receptor agonist” AND “bunionectomy”; “µ-opioid receptor agonist” AND “hallux valgus surgery”; “µ-opioid receptor agonist” AND “orthopedic surgery”; “noradrenaline reuptake inhibitor” AND “bunionectomy”; “noradrenaline reuptake inhibitor” AND “hallux valgus surgery”; “noradrenaline reuptake inhibitor” AND “orthopedic surgery”; “opioid” AND “bunionectomy”; “opioid” AND “hallux valgus surgery”; and “opioid” AND “orthopedic surgery”. On the other hand, a single-term search was used for the Imbiomed database. Filters for study type/design and language (“English” and “Spanish”) were used. This activity was performed by two independent researchers. The protocol was registered in the International Prospective Register of Systematic Reviews (PROSPERO ID CRD42023437295) of the National Institute of Health and Care Research at the University of York. Articles published up to 30 May 2023, were eligible.

### 2.2. Population, Interventions, Control, and Outcome Strategy (Inclusion Criteria)

Population: Patients undergoing a bunionectomy.

Interventions: Administration of tapentadol IR.

Control: Placebo.

Outcome: Sum of pain intensity (SPID), total pain relief (TOTPAR), global evaluation, and adverse effects.

### 2.3. Exclusion Criteria

Clinical studies reporting a loss to follow-up of more than 20%.

High risk of bias according to the Cochrane Collaboration risk of bias tool.

### 2.4. Assessment of Bias

Quality assessment of each clinical assay was performed with the Cochrane Collaboration risk of bias tool [13,14,15]. The studies without a high risk of bias were deliberated like of high quality. Two independent researchers made a full evaluation of each report and discussed their differences to obtain a consensus [13,14,15,16,17].

### 2.5. Data Extraction

Extracted data were as follows: author, design study, treatment groups, size sample (n), dose, SPID, TOTPAR, adverse effects on the gastrointestinal tract (nausea, vomiting, constipation), and adverse effects on the nervous system (dizziness, somnolence, and headache).

### 2.6. Statistical Analysis

Inverse variance statistical methods with means difference were used to assess the numerical data. The Mantel–Haenszel test and the Odd Ratio (OR) were utilized to analyze dichotomous data. In order to follow a conservative statistical method, all meta-analyzes were made with a random effect model employing the Review Manager Software 5.3 for Windows. In addition, inconsistency or heterogeneity was assessed using the I^2^ value in each meta-analysis. Low heterogeneity was considered when the I^2^ value was between 0% and 30%, moderate heterogeneity when the I^2^ value was between 31% and 60%, and high heterogeneity when the I^2^ value was between 61% and 100%. A *p*-value overall test <0.05 and an OR (>1 and within the 95% confidence interval (CI)) deliberated a statistical difference [14,18,19,20]. 

The number needed to treat (NNT), the number needed to harm (NNH), and the 95% CI were calculated for the global evaluation and adverse effects using the Risk Reduction Calculator from the University of Illinois at Chicago [21].

## 3. Results

### 3.1. Information Search and Bias

**Figure 1:**
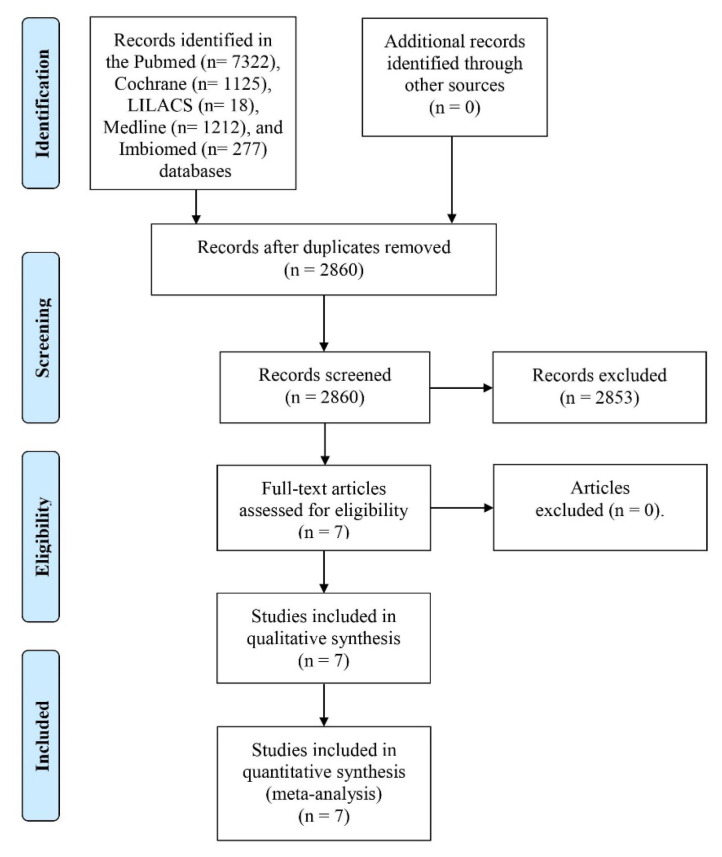
Study flow diagram.

**Figure 2:**
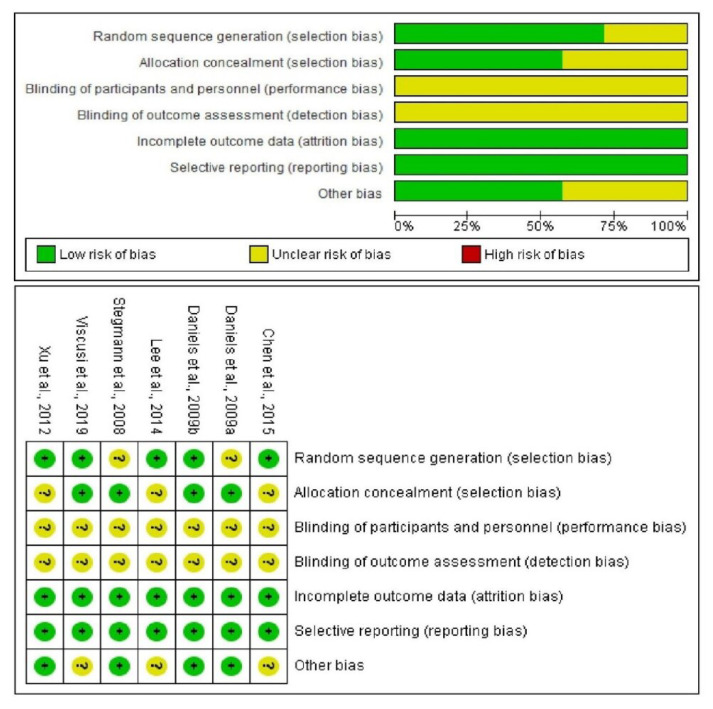
Assessment of risk of bias [6,7,8,9,10,11,12]. + = Low risk of bias? = Unclear risk of bias.

A total of 2860 abstract publications were identified in the databases used, of which only 7 abstracts were considered for full-text evaluation. These articles were then fully assessed using the PICO criteria and the Cochrane Bias Assessment Tool. These same seven articles were considered for the qualitative evaluation, and six were considered for the statistical analysis (Figure 1 and Figure 2) [6,7,8,9,10,11,12].

### 3.2. Qualitative Assessment

A total of seven studies were included in the qualitative evaluation (*n* = 2381). According to the authors’ conclusions, tapentadol (*n* = 1772) was more effective in relieving postoperative pain than the placebo (*n* = 609) after a bunionectomy (Table 1).

### 3.3. Quantitative Evaluation

**Figure 3:**
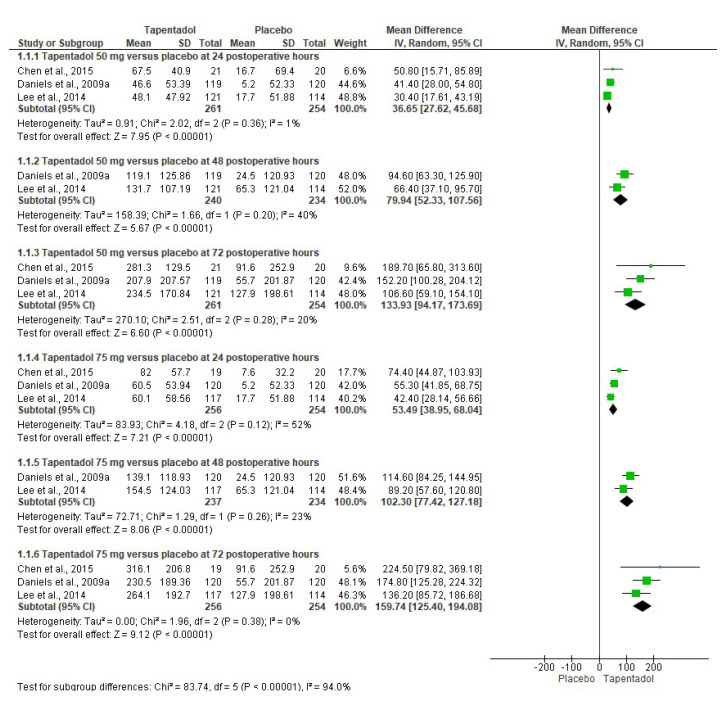
Pooled data analysis of SPID [6,7,9].

The pooled analysis of the SPID endpoint comparing tapentadol IR versus the placebo was performed using three clinical trials. Tapentadol IR 50 mg (*n* = 261) and tapentadol IR 75 mg (*n* = 256) were more effective for postoperative pain control than the placebo (*n* = 254) at 24, 48, and 72 postoperative hours (Figure 3) [6,7,9]. In addition, the uncertainty, assessed through the heterogeneity of the data, showed mainly low heterogeneity; however, two time points showed moderate heterogeneity, so we decided to use a random effects model (Figure 3) [6,7,9]. 

**Figure 4:**
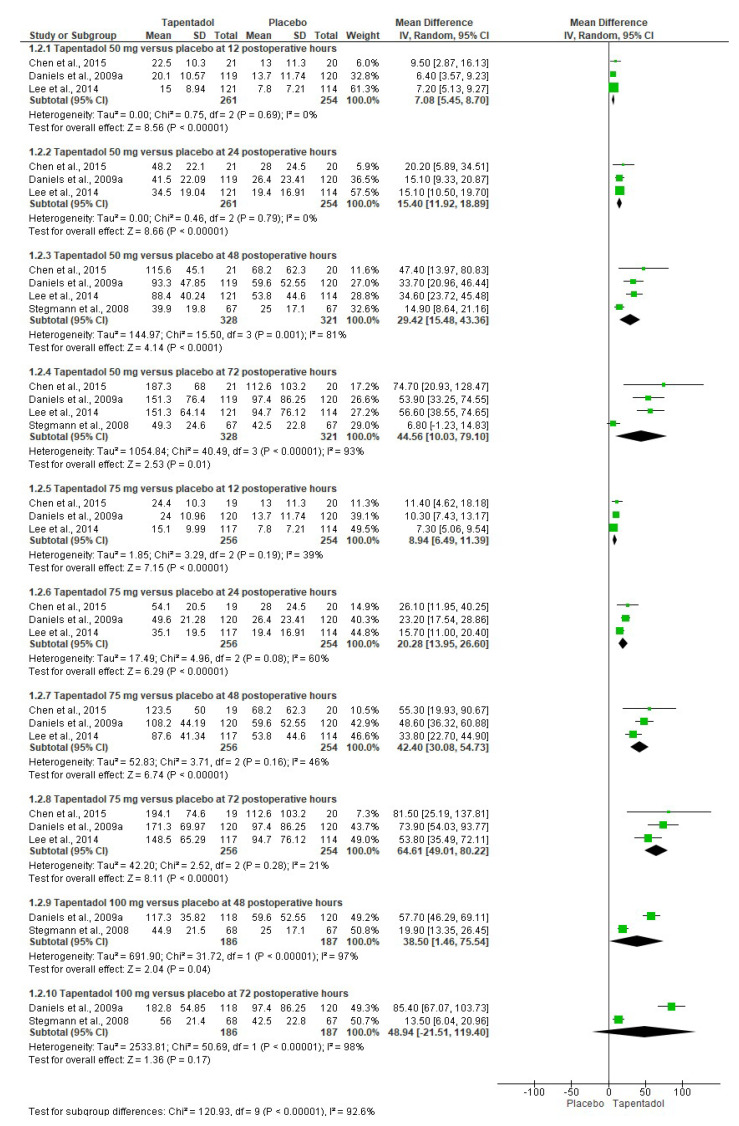
Meta-analysis of TOTPAR [6,7,9,10].

The TOTPAR of tapentadol IR and the placebo were evaluated using data from four studies. The results show that the three doses of tapentadol IR—50 mg (*n* = 328), 75 mg (*n* = 256), and 100 mg (*n* = 186)—presented better analgesic efficacy than the placebo (*n* = 321, *n* = 254, and *n* = 187, respectively) at 12, 24, 48, and 72 postoperative hours (Figure 4) [6,7,9,10]. The evaluation of the data showed that the clinical indicator of analgesia TOTPAR mainly presented a high heterogeneity. For this reason, the data analysis was performed with a conservative approach using a random effects model (Figure 4) [6,7,9,10].

**Figure 5:**
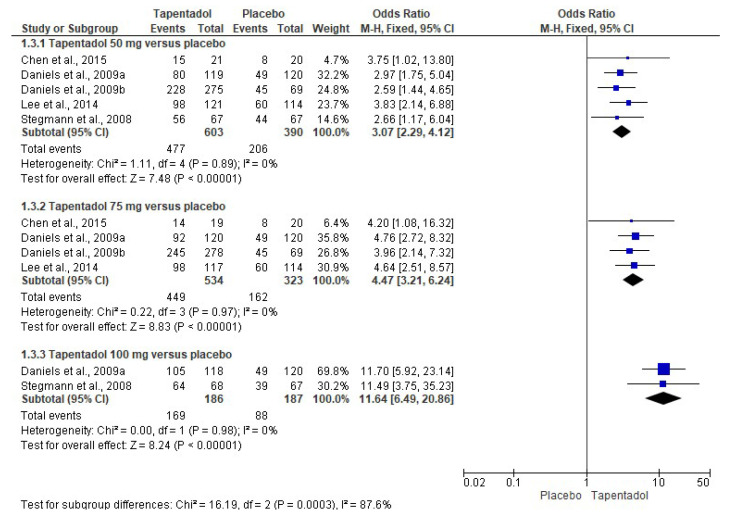
Combined analysis of the global evaluation [6,7,8,9,10].

The global evaluation of the treatments (patient satisfaction) was carried out with five clinical trials and showed that any dose of tapentadol IR—50 mg (*n* = 603), 75 mg (*n* = 534), and 100 mg (*n* = 186)—was better evaluated by the patients compared to the placebo (*n* = 390, *n* = 323, and *n* = 187, respectively) (Figure 5) [6,7,8,9,10]. In addition, the NNT shows that 3.8 (95% CI = 3.1 to 4.9) patients must be treated with tapentadol IR 50 mg to obtain one analgesic success that would not have occurred with the placebo. In this sense, the NNT for tapentadol IR 75 mg and tapentadol IR 100 mg was 2.9 (95% CI = 2.5 to 3.6) and 2.2 (95% CI = 1.8 to 2.7) in comparison with the placebo, respectively. Statistical analysis of the global assessment data showed low heterogeneity, so the fixed effects model was used (Figure 5) [6,7,8,9,10].

### 3.4. Adverse Effects

**Figure 6:**
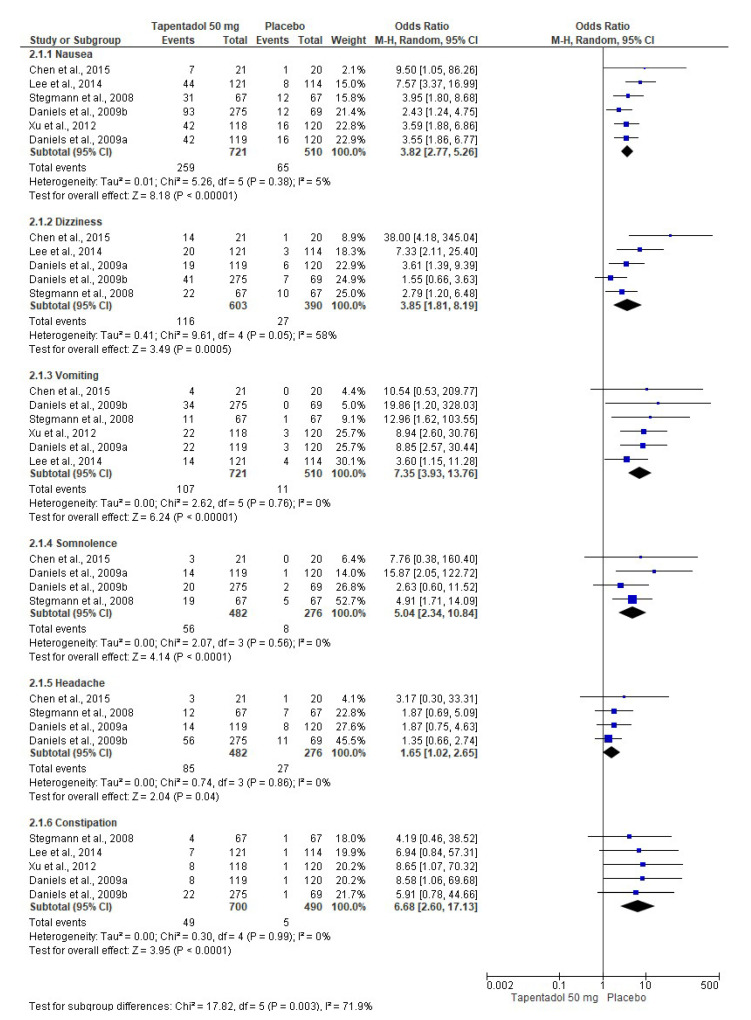
Meta-analysis of the adverse effects of 50 mg of tapentadol [6,7,8,9,10,12].

**Figure 7:**
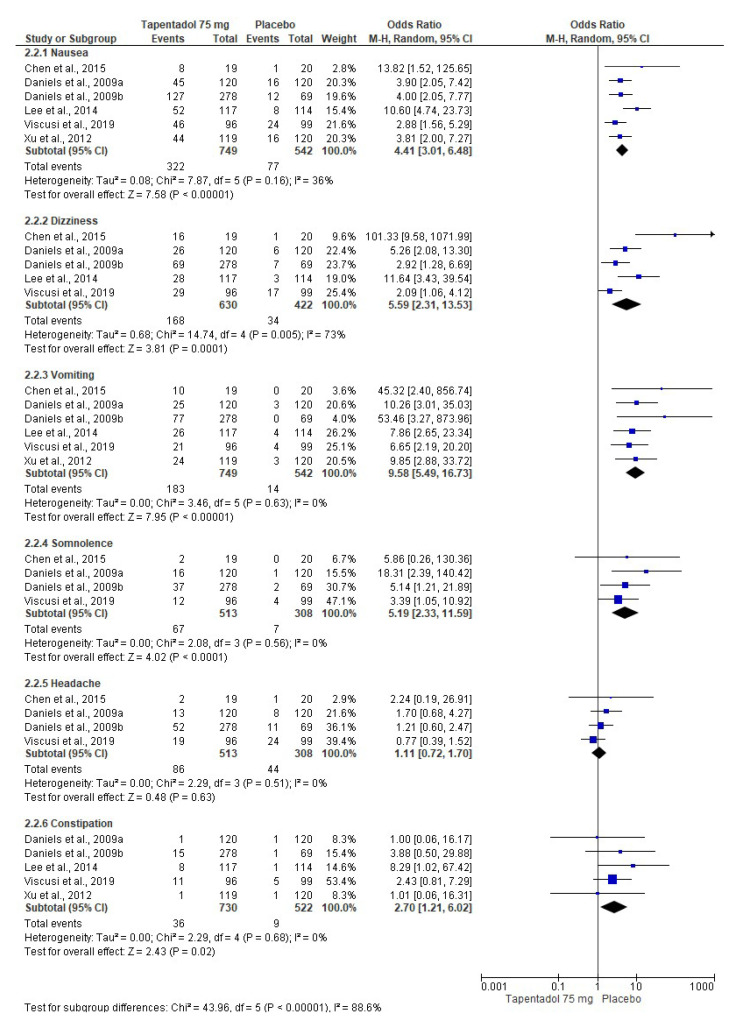
Combined analysis of the adverse effects of 75 mg of tapentadol [6,7,8,9,11,12].

**Figure 8:**
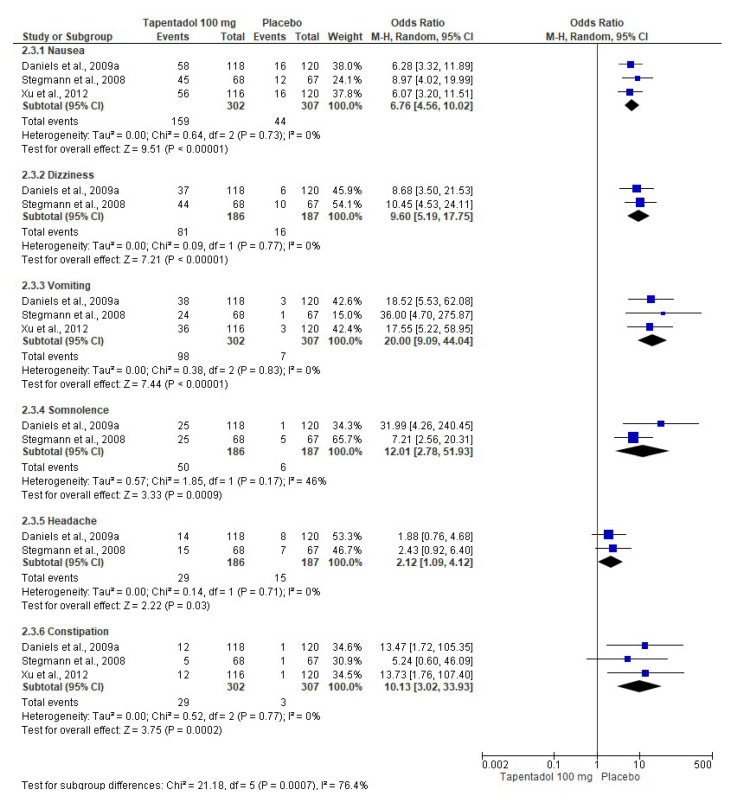
Pooled data analysis of the adverse effects of 100 mg of tapentadol [7,10,12].

The pooled evaluation of the adverse effects of tapentadol IR was performed with seven clinical trials. In this sense, tapentadol IR (50 mg (*n* = 721, Figure 6) [6,7,8,9,10,12], 75 mg (*n* = 749, Figure 7) [6,7,8,9,11,12], and 100 mg (*n* = 302, Figure 8) [7,10,12]) produced a high number of adverse effects on the nervous system and gastrointestinal tract compared to the placebo (*n* = 510, Figure 6; *n* = 542, Figure 7; and *n* = 307, Figure 8). Table 2 shows the NNH and 95% CI of the adverse effects of tapentadol IR compared to the placebo. The evaluation of the adverse effects of the three doses of tapentadol IR used showed high heterogeneity (at least in one adverse effect evaluated), so it was decided to carry out the three meta-analyses using the random effects model.

## 4. Discussion

To our knowledge, this is the first systematic review to use data from other studies to perform a statistical analysis to determine the dose with the best analgesic efficacy and tolerability of tapentadol IR in a kind of specific surgery, a bunionectomy. The meta-analyses performed showed that the analgesic efficacy of tapentadol IR is dose-dependent; that is, patients reported better pain relief with higher doses. In the same way, the meta-analyses demonstrated that the adverse effects also depend on the dose of tapentadol IR; that is, the higher the dose used, the greater the number of patients reported adverse effects.

According to the Cochrane bias assessment tool, the risk of bias in the studies included in this study was low to moderate. Items from the Cochrane bias assessment tool least likely to be biased were incomplete data reporting and selective reporting, while studies most likely to be biased were those that made reference to blinding. In all cases, the specifications of who was blinded were not included in the articles, and only the term double-blind was included, so these terms were classified as unclear risk of bias (Figure 2).

Qualitative analysis showed that tapentadol IR produces better postoperative pain relief compared to a placebo after bunion surgery. Through the characteristics of each study included in this systematic review and meta-analysis, a standardized surgical procedure can be observed, minimizing possible biases due to differences in the execution of the surgery (Table 1).

The analgesic efficacy of tapentadol IR (*n* = 1323) compared to the placebo (*n* = 390) was evaluated in a total of 1713 patients through a global evaluation of the treatments. All three doses of tapentadol IR showed better results compared to the placebo after the bunionectomy. Based on the NNT, 100 mg of tapentadol could be considered the most effective dose for postoperative pain control, as it had the lowest NNT = 2.2 (95% CI = 1.8 to 2.7) compared to tapentadol IR 50 mg NNT = 3.8 (95% CI = 3.1 to 4.9) and tapentadol IR 75 mg NNT = 2.9 (95% CI = 2.5 to 3.6). However, the statistical analysis of tapentadol IR 100 mg versus the placebo had a smaller sample size (*n* = 373) compared to tapentadol IR 50 mg (*n* = 993) and tapentadol IR 75 mg (*n* = 857). It is important to note that the global evaluation of the treatments was the most powerful analgesic indicator to determine the analgesic efficacy of the three doses of tapentadol IR because pooled data analysis and NNT calculations were possible. Likewise, it is important to emphasize that the heterogeneity of this meta-analysis was null in the three comparisons that were made, which allowed the use of the fixed effects method and greater power in the data analysis.

A systematic review and meta-analysis to assess the analgesic efficacy of tapentadol in different types of surgery have been previously reported. Xiao et al., 2017 [22], performed a meta-analysis to assess the analgesic efficacy and safety of tapentadol in a bunionectomy, a hysterectomy, end-stage joint disease, and joint replacement surgery. This study compared the analgesic efficacy of tapentadol versus a placebo using the SPID clinical indicator at 48 h post-surgery, demonstrating that doses of 50 mg, 75 mg, and 100 mg of this drug are better than a placebo for controlling postoperative pain by the evaluation of pooled data obtained in studies with these different types of surgery. Likewise, this study compared the analgesic efficacy of tapentadol at doses of 50 mg, 75 mg, and 100 mg versus oxycodone at doses of 10 mg and 15 mg. In all cases where a cluster analysis is shown to compare these two analgesics, *p*-values with no statistical difference were obtained. Therefore, we could conclude that these drugs might have similar analgesic efficacy, indicating a problem, with the different types of surgery included in this statistical analysis. Our study only included studies that reported the analgesic efficacy of tapentadol IR compared to a placebo in a bunionectomy using three indicators of analgesic efficacy: SPID, TOTPAR, and global assessment of analgesics.

A total of 2381 participants were included in the evaluation of the adverse effects of tapentadol IR (*n* = 1772) in comparison to the placebo (*n* = 609) in a bunionectomy. Like the analgesic effect of tapentadol IR, the adverse effects have a direct relationship depending on the dose, and tapentadol IR 100 mg was the dose that presented the greatest number of adverse effects. According to the NNH, it is necessary to administer tapentadol IR 100 mg to 2.5 patients for 1 patient to experience nausea, while for this to happen with tapentadol IR 75 mg and tapentadol IR 50 mg, it is necessary that 3.4 and 4.3 patients receive tapentadol, respectively.

Currently, international organizations support the use of multimodal analgesia to treat postoperative pain [23]. The use of a combination of analgesics (NSAIDs, glucocorticoids, and opioids) could have many advantages, such as the induction of analgesia by different mechanisms of action, improved rapid recovery after surgery, less general toxicity, and adverse effects because the combination of drugs allows reducing the doses, particularly how much synergistic analgesic effects can be obtained using a combination of two or more drugs [24,25,26,27,28,29,30]. It is important to mention that evidence can also be found in clinical trials on the use of other opioid analgesics alone, or in combination with other types of drugs, to treat postoperative pain after a bunionectomy, such as tramadol [31,32], oxycodone [7,33,34,35], and morphine [33,36,37].

Several systematic reviews and meta-analyses have been performed to assess both the analgesic efficacy and adverse effects of tapentadol in different areas of medicine. Etropolski et al., 2014, conducted a systematic review and meta-analysis to assess the adverse effects of a placebo, oxycodone, and tapentadol for the treatment of pain in osteoarthritis and lower back pain. Quantitative results show that the placebo was safer than both drugs, while tapentadol had fewer adverse effects on the gastrointestinal tract and central nervous system compared with oxycodone [38]. Santos et al., 2015, conducted a systematic review and meta-analysis to assess the efficacy and adverse effects of a placebo, oxycodone, and tapentadol in musculoskeletal pain. The results of this study showed a lower incidence of adverse effects of the placebo compared to both drugs, while tapentadol showed a better safety profile than oxycodone [39]. Wiffen et al., 2015, reported that tapentadol, morphine, and oxycodone had similar safety profiles or adverse effects in studies conducted for cancer pain relief [40]. Xiao et al., 2016, demonstrated that tapentadol IR 50 mg and 75 mg produced fewer adverse effects compared to oxycodone when these drugs were administered to patients undergoing different surgical procedures [22]. Meng et al., 2017, evaluated the prevalence of adverse effects of different opioid analgesics and reported that tapentadol had the lowest number of adverse effects [41]. In our systematic review and meta-analysis, the adverse effects of tapentadol on the central nervous system and the gastrointestinal tract were included in our pooled evaluation. All comparisons showed that tapentadol has a higher number of adverse effects compared to the placebo. However, considering a risk–benefit ratio, it could be considered that the analgesic efficacy of tapentadol is greater than the risk of adverse effects. It is important to note that serious adverse effects were not reported in any of the studies included in this systematic review and meta-analysis.

This is the first systematic review that analyzes pooled data to determine the analgesic efficacy and adverse effects of IR tapentadol after a bunionectomy, achieving the largest sample size reported to date in this surgical area. In this sense, it is important to mention that the meta-analyses carried out in this study present highly variable heterogeneity (low, moderate, and high heterogeneity). On the other hand, this study allows us to know the efficacy of tapentadol for the control of postoperative pain in patients with a bunionectomy, which was achieved thanks to the collection of available information to perform statistical calculations (NNT and NND) that can be applied by the physician in the care of the patients. In both cases—the evaluation of efficacy and adverse effects—it was possible to calculate the NNT and NNH, which adds clinical importance to the use of tapentadol in this surgical area and that if there are other similar studies that evaluate other analgesics, could help decide which medication might be the best treatment option. Furthermore, it is important to note that only studies with low or unclear risk of bias according to the Cochrane tool were included in this systematic review and meta-analysis [42,43,44]. These represent the advantages of our study. On the other hand, the main drawback of this study stems from its retrospective design and publication bias [42,43,44].

Based on the global evaluation of patient satisfaction, it is concluded that the 100 mg dose of tapentadol IR is the one that obtained the best satisfaction score. This could be interpreted as patients experiencing less pain during the postoperative evaluation period and fewer adverse effects, which would make this dose of tapentadol IR the most clinically effective for treating postoperative pain in patients undergoing bunion surgery.

## Figures and Tables

**Table 1:** Characteristics of the included studies.

ID Study and Study Design	Treatments (*n*)	Details of Patients, Surgical Procedure, and Evaluation	Authors’ Conclusion
Chen et al., 2015 [6]. Phase 3, randomized, double-blind, placebo-controlled, parallel-group, multicenter clinical trial.	Group A: Tapentadol IR 50 mg (*n* = 21). Group B: Tapentadol IR 75 mg (*n* = 19). Group C: Placebo (*n* = 20). The treatments were given orally every 4–6 h over a 72 h period.	Healthy or medically stable patients aged 20 to 80 years needing a first metatarsal bunionectomy were included. The bunionectomy was made using standardized surgical procedures. The continuous popliteal sciatic block was used. The taking of drugs that interfered with the perception of postoperative pain, such as opioid analgesics or other analgesic or sedative medications, was not allowed during the treatment phase. Subjects who took a different drug than the study drug were discontinued from the clinical trial because of a lack of analgesic efficacy. Pain intensity, pain relief, patient global assessment, SPID, TOTPAR, pain intensity difference (SPRID), and adverse effects were evaluated.	Tapentadol relieved moderate to severe acute pain and had an acceptable safety profile compared to the placebo.
Daniels et al., 2009 [7]. Phase 3, randomized, double-blind, placebo-and-active-controlled, parallel-group, multicenter clinical assay.	Group A: Tapentadol IR 50 mg (*n* = 119). Group B: Tapentadol IR 75 mg (*n* = 120). Group C: Tapentadol IR 100 mg (*n* = 118). Group D: Oxycodone HCl IR 15 mg (*n* = 125). Group E: Placebo (*n* = 120). The treatments were given orally every 4–6 h over a 72 h period.	ASA-1-to-3 patients aged 18 to 80 years who had a pain score ≥ 4 after the bunionectomy were eligible. The continuous popliteal sciatic block was used. Rescue medication with acetaminophen, ketorolac, and/or hydrocodone/acetaminophen combination was allowed. SPID, TOTPAR, patient global evaluation, and adverse effects were evaluated.	Tapentadol had better analgesic efficacy and lower adverse effects when compared to the placebo. Tapentadol 100 mg had similar analgesic activity to 15 mg of oxycodone.
Daniels et al., 2009 [8]. Phase 3, randomized, double-blind, placebo-and-active-controlled, parallel-group, multicenter study.	Group A: Tapentadol IR 50 mg (*n* = 275). Group B: Tapentadol IR 75 mg (*n* = 278). Group C: Oxycodone HCl IR 10 mg (*n* = 278). Group D: Placebo (*n* = 69). The treatments were given orally every 4–6 h over a 72 h period.	ASA-1 and ASA-2 patients aged 18 to 80 years who had a pain score ≥ 4 after the bunionectomy were eligible. The continuous popliteal sciatic block was used. Patients were allowed up to 2 g of acetaminophen after receipt of the first dose of study medication. SPID, TOTPAR, patient global evaluation, and adverse effects were evaluated.	Tapentadol relieved moderate to severe acute pain and had an acceptable safety profile compared to the placebo. Furthermore, both doses of tapentadol were not inferior to oxycodone.
Lee et al., 2014 [9]. Phase 3, randomized, double-blind, placebo-controlled, parallel-group, multicenter clinical trial.	Group A: Tapentadol IR 50 mg (*n* = 121). Group B: Tapentadol IR 75 mg (*n* = 117). Group C: Placebo (*n* = 114). The treatments were given orally every 4–6 h over a 72 h period.	Healthy or medically stable patients aged 20 to 80 years needing a first metatarsal bunionectomy were included. The 0.5% mepivacaine continuous popliteal sciatic block was used. Acetaminophen at a dose of 650 mg taken orally (PO) and/or ketorolac at a dose of 30 mg IV every 4–6 h, or alternatively fentanyl at a dose of 100 μg IV were used as supplemental analgesia until the start of the qualifying period. SPID, TOTPAR, SPRID, global evaluation, and adverse effects were evaluated.	Tapentadol relieved the acute pain when compared to the placebo.
Stegmann et al., 2008 [10]. Phase II, randomized, double-blind, placebo-controlled, parallel-group, multiple-dose clinical assay.	Group A: Tapentadol IR 50 mg (*n* = 67). Group B: Tapentadol IR 100 mg (*n* = 68). Group C: Oxycodone HCl IR 10 mg (*n* = 67). Group D: Placebo (*n* = 67). The treatments were given orally every 4–6 h over a 72 h period.	Healthy or medically stable patients aged 18 to 65 years needing a first metatarsal bunionectomy were included. The 0.5% mepivacaine sciatic block was used. The use of analgesics, sedatives, and narcotic drugs 12 h before surgery was not allowed. Ibuprofen, ketorolac, and the hydrocodone–acetaminophen combination as rescue medication were used. SPID, TOTPAR, analgesic consumption, global evaluation, and adverse effects were evaluated.	Both tapentadol and oxycodone were effective in postoperative pain control.
Viscusi et al., 2019 [11]. Phase 3, randomized, double-blind, placebo-and-active-controlled, parallel-group, multicenter trial.	Group A: Tapentadol IR 75 mg (*n* = 96). Group B: Morphine 30 mg (*n* = 96). Group C: Placebo (*n* = 99). The treatments were given orally every 4–6 h over a 72 h period.	Patients aged 18 to 65 years needing a first metatarsal bunionectomy were included. The 0.5% mepivacaine continuous popliteal sciatic block was used. Paracetamol–hydrocodone combination rescue medication was used. SPID, the first intake of the investigational medicinal product, global evaluation, and adverse effects were evaluated.	Tapentadol was effective in postoperative pain control and was well-tolerated.
Xu et al., 2012 [12]. Randomized, double-blind, placebo-and-active-controlled, parallel-group, clinical trial.	Group A: Tapentadol IR 50 mg (*n* = 118). Group B: Tapentadol IR 75 mg (*n* = 119). Group C: Tapentadol IR 100 mg (*n* = 116). Group D: Oxycodone HCl IR 15 mg (*n* = 123). Group E: Placebo (*n* = 120). The treatments were given orally every 4–6 h over a 72 h period.	Healthy patients with moderate to severe pain following the bunionectomy. Data for regional anesthesia and rescue medication were not included. The adverse effects—nausea, vomiting, and constipation—were evaluated.	A decrease in the adverse effects of tapentadol compared to oxycodone was observed.

**Table 2:** The NNH and 95% IC for tapentadol when compared to the placebo.

	Tapentadol 50 mg		Tapentadol 75 mg		Tapentadol 100 mg	
	NNH	95% CI	NNH	95% CI	NNH	95% CI
Nausea	4.3	3.5 to 5.4	3.4	2.9 to 4.1	2.5	2 to 3.2
Dizziness	8.1	6.1 to 12.1	5.4	4.4 to 7	2.9	2.3 to 3.7
Vomiting	8.3	6.6 to 11.2	4.4	3.8 to 5.3	3.2	2.6 to 4.1
Somnolence	11.5	8.2 to 19.1	9.3	7.1 to 13.5	4.2	3.3 to 5.9
Headache	12.7	7.9 to 33.7	40.3	13.2 to infinity	13.2	7.1 to 94.1
Constipation	16.8	12.1 to 27.5	26.8	16.6 to 69.2	12.4	8 to 27.2

## Data Availability

Data supporting the findings of this study are available in each included article, and pooled data are contained in this article.
